# A Comparative Study of the Effect of Commonly Used Pesticides on Cervical Contractions in Pregnant Cows, In Vitro

**DOI:** 10.3390/toxics13090793

**Published:** 2025-09-17

**Authors:** Michal Hubert Wrobel

**Affiliations:** Institute of Animal Reproduction and Food Research, Polish Academy of Sciences, 10-683 Olsztyn, Poland; m.wrobel@pan.olsztyn.pl

**Keywords:** pesticides, insecticides, herbicides, contractions, cervix, pregnancy, cow

## Abstract

Organochlorine insecticides (DDTs), organophosphate insecticides (malathion), carbamate insecticides (carbaryl and thiram), pyrethroid (cypermethrin and fenvalarate) insecticides, and herbicides (glyphosate and atrazine) were selected for this study because they disrupt cervical and myometrial function in the bovine oestrous cycle. However, their potential to affect reproductive success in cattle during pregnancy has not been directly confirmed. The aim of this study was to determine the effects of the investigated pesticides on cervical contractions in pregnant cows. Cervical strips from cows at 4–6 months of gestation were treated with the eight singular pesticides (used at non-toxic, environmental dose) or oestradiol (E2) under two different conditions (37.5 °C for 24 h and 4 °C for 48 h), which were applied to assess pesticide effects under both physiological and prolonged-exposure settings. The strength of the contractions was then measured. The findings of the study demonstrated that both the carbamates and glyphosate increased the force of cervical strip contractions to a greater extent than cypermethrin. In contrast, fenvalerate was observed to induce a state of cervical relaxation, analogous to the effects of E2, while DDT, malathion and atrazine exerted no effect on cervical motor function during the period of pregnancy under investigation. These preliminary findings indicate a potential impact of pesticides on cervical function during pregnancy, but should be interpreted with caution as they are based on isolated tissue at a single concentration. Further in vivo and dose–response studies are needed to confirm their biological and clinical relevance.

## 1. Introduction

Plant protection products find application in the management of pests, which may include insects and weeds. The predominant chemical classifications of synthetic insecticides encompass the categories of organochlorine, organophosphate, carbamate, and pyrethroid. Following the production of DDT in the late 1940s, organochlorine chemicals were the first to be introduced. The recognition of DDT as a persistent environmental pollutant [1] has led to its prohibition in many Western countries [2]. However, DDT is still used in developing countries in Africa and Asia for the control of malaria [3,4]. Subsequently, during the 1960s, organochlorine insecticides were replaced by organophosphates such as malathion, and since 1970, carbamates have been extensively used [5]. It is acknowledged that carbaryl, the most prevalent of these, is a broad-spectrum insecticide, while thiram is extensively utilised in agriculture as a fungicide [6,7]. Today, pyrethroids are among the most widely used insecticides worldwide, with over 3500 products registered since the 1980s. They were poised to replace organophosphates and possibly at least some carbamates in the insecticide market [5,8]. On the other hand, the non-selective, post-emergence, systemic herbicide glyphosate dominated the global herbicide market, and atrazine, frequently used in combination with glyphosate, remains a staple of American and Asian agriculture [9,10,11].

Due to their persistence and widespread use, all the pesticides studied have been detected in the environment or in living organisms. Furthermore, these levels have been shown to be highly comparable. Carbaryl has been detected in water (0.2–4 ng/mL) [12] and soil (1.3–1.67 ng/mL) [13]. Theses concentrations are included within the range of 0.02–20 ng/mL of atrazine reported as its environmentally relevant level [14], while malathion has been detected in water samples at concentrations ranging from 10 to 100 ng/mL [15]. Furthermore, glyphosate levels in human serum range from 0.2 to 189 ng/mL [16], while 0.25 ng/mL and 0.6–11 ng/mL of DDT were measured in bovine follicular fluid [17] and human milk [18], respectively. However, studies have reported concentrations of cypermethrin and fenvalerate in bovine and human milk ranging from 0.03 to 4.24 ng/mL and 0.09–0.17 ng/mL, respectively [19], while their urinary metabolites have been measured in the range of 0.4–8.85 ng/mL and cypermethrin even reaching 151 ng/mL in human blood [20,21].

After bioaccumulation, these pesticides have the potential to act as endocrine disruptors [22] and their presence is not indifferent to health, including reproduction [23]. Consequently, particular attention should be paid to the association between some of them and an increased risk of miscarriage or premature birth. This association has been the subject of previous studies, albeit not always with definitive confirmation [24,25,26,27,28,29,30,31,32]. It has also been suggested that a significantly lower pregnancy rate or live birth rate cannot be excluded [33]. In fact, the occurrence of pregnancy losses is a salient issue, as evidenced by the substantial body of literature on the subject [34]. This particular phenomenon serves as a quantifiable metric, offering a tangible illustration of the disruptive effect on reproductive processes.

The cervix plays a pivotal role during pregnancy by maintaining the foetus in a safe and contained uterine environment that gradually closes. In addition, it has been demonstrated that the substance under discussion has the capacity to act as an agent that obstructs the ascent of pathogens from the vagina into the uterine cavity [35]. It is imperative to note that, in order to facilitate the transportation of sperm following sexual intercourse, the cervix must be in a state of openness [36,37]. Moreover, the process of dilation occurs during labour, a phenomenon facilitated by the contraction of the uterus. This results in the creation of a passageway for the foetus to emerge into the world [38]. It has been established that successful delivery is contingent upon the optimal timing of cervical ripening and dilation, in conjunction with myometrial contractions [38]. It is noteworthy that in certain instances of premature cervical remodelling, the internal os (in proximity to the uterus) is the primary site of weakness. It is also necessary to point out that changes only at myometrial contractions without the disturbance of the cervical activity may not be sufficient to cause the preterm delivery, while cervical incompetence leading to premature labour is considered as one of the main causes of prenatal mortality [39,40]. From this standpoint, it is imperative to acknowledge the significance of comprehending the alterations in the regulatory mechanisms of cervical activity in the context of environmental pollutants. This understanding is of paramount importance from both a cognitive and a practical standpoint, as well as from an economic perspective. To date, the effect of DDT on the motor activity of bovine smooth muscle from the uterine horn during pregnancy has been the sole subject of study [41].

The cervix is defined as a firm cylinder, connecting the vagina (external os) and the uterus (internal os). Despite the anatomical disparity between the cervix and the uterus, both structures are interconnected via direct anatomical connections, such as gap junctions, which facilitate direct communication between these two anatomical structures. The cervix is lined with a mucus-secreting epithelium, while the deeper part is composed of extracellular matrix (up to 90%, mostly collagen, some elastin, proteoglycans) with minimal cellular content (10–15% fibroblasts, smooth muscle cells, glandular cells, vascular cells, immune cells). The cervix is not a homogeneous structure. A notable observation is the presence of significant collagen cross-linking heterogeneity between the internal and external os. The orientation of the inner and outer muscle layers is circular and longitudinal, respectively. The longitudinal muscle layer of the cervix is thicker than the circular layer, and the area of the internal os is significantly more cellular than of the external os, containing approximately 50–60% smooth muscle cells. The outer smooth muscle layer of the bovine cervix is also comparatively thick, in comparison to other species. In addition, the muscle layer on the uterine side is much thicker than that of the vaginal os. [38,39,40,42]. The outer cervical layer of smooth muscle layer has been described as continuous with the outer myometrial layer, but they are regulated differently (in bovine tissue [43]) and thus can function independently. It can be concluded that contractions of the cervix are interrelated, coordinated, and independent of contractions of the myometrium. Furthermore, the cervix and the uterine horn exhibit differential susceptibility to regulators of contraction [44,45,46]. Research has shown that oestradiol (E2) induces the production of prostaglandins, which ultimately alter myometrial and cervical tissues and thereby also leads to relaxation of the cervix via its dilation or ripening at any stage of pregnancy [47,48]. As demonstrated in numerous studies, the estrogenic properties of DDT [49,50], carbaryl [51], cypermethrin [52], glyphosate [53,54], fenvalerate [55], malathion [56] have been well documented. Furthermore, atrazine has been demonstrated to induce both, estrogenic [57] and antiestrogenic properties [58], and it has been suggested that thiram may elicit a similar effect [59]. This is the point at which risk to uterine function can be seen, but disruption of only one element may not be sufficient to cause preterm birth. Therefore, the effects on both parts of the uterus must be studied and compared individually. A number of studies have previously carefully investigated the effect of environmental doses of pesticides on myometrial [60,61,62,63] and cervical [59,64,65,66] strips from cows during the oestrous cycle. According to the findings of the studies, the most effective dose of pesticides was then selected.

The objective of this study was to ascertain the impact of the pesticides under investigation on cervical contractions during pregnancy. The measurement was conducted with the objective of estimating the potential to disrupt the final reproductive success. Furthermore, in accordance with the findings of our preceding studies concerning the impact of xenobiotics on myometrial contractions, the second objective of these studies was to ascertain the conditions of cervical incubation with the most protracted treatments of environmental doses.

## 2. Materials and Methods

### 2.1. Chemicals

Atrazine: 2-chloro-4-ethylamino-6-isopropylamino-1,3,5–triazine [CAS No. 1912-24-9]; carbaryl: 1-Naphthyl-N-methylcarbamate [CAS No. 63-25-2]; cypermethrin: [cyano-(3-phenoxyphenyl)methyl] 3-(2,2-dichloroethenyl)-2,2-dimethylcyclopropane-1-carboxylate [CAS No. 52315-07-8]; DDT: 1,1,1-Trichloro-2,2-bis (4-chlorophenyl)ethane [CAS No. 50-29-3]; fenvalerate: α-Cyano-3-phenoxybenzyl α-(4-chlorophenyl)-iso-valerate [CAS No. 51630-58-1]; glyphosate: N-(phosphonomethyl) glycine [CAS No. 1071-83-6]; malathion: diethyl 2-dimethoxyphosphinothioylsulfanylbutanedioate [CAS No. 121-75-5]; and thiram (bis-dimethylthiocarbamoyl disulfide) [CAS No. 137-26-8]. All pesticides were of analytical standards purity. They were dissolved in DMSO (HPLC purity grade) at the doses studied, which were in the environmentally relevant range (10 ng/mL). The final concentration of DMSO in the culture media did not exceed 0.1%. Therefore, 0.1% DMSO was added to the control samples. Each medium was then supplemented with gentamicin (20 μL/mL). All materials used in these studies were purchased from Sigma-Aldrich (Poznan, Poland) unless otherwise stated.

### 2.2. Material

Bovine cervixes were obtained from a commercial abattoir at 4–6 months of gestation. The stage of gestation was determined according to the criteria of Hafez [67]. Cervical strips (5–6 mm in length) were prepared for the study of the longitudinal smooth muscle layers. The strips were immersed in 2 mL of physiological salt solution (116 mM NaCl, 4.6 mM KCl, 1.16 mM NaH_2_PO_4_-H_2_O, 1.16 mM MgSO_4_ × 7H_2_O, 21.9 mM NaHCO_3_, 1.8 mM CaCl_2_-2H_2_O, 11.6 mM dextrose and 0.03 mM CaNaEDTA; pH = 7.4) and aerated (95% air and 5% CO_2_) as previously described [59,64,65,66]. The strips were treated as described in the experiment, with half being incubated for 24 or 48 h at 37.5 °C or 4 °C, respectively. In this case, the low temperature used was verified according to our previously tested model [60,61,63]. Cervical cells were obtained for preliminary studies only, to confirm that the studied dose of pesticides did not induce a cytotoxic effect. They were obtained by enzymatic dispersion according to Wrobel and Mlynarczuk [59] and Wrobel et al. [64,65,66]. For this purpose, 7 g of tissue were minced and then digested for 2 h in warm (38 °C) and oxygenated (95% O_2_ + 5% CO_2_) medium (M199 + 0.1% BSA; 20 mL) supplemented with dispase (0.2 mg/mL Gibco, Glasgow, UK) and collagenase IA (1.5 mg/mL). Only cells with a viability greater than 80%, as assessed by trypan blue staining, were used for further studies. The cells were then suspended in DMEM/HAM-12 supplemented with 5% FCS (5 × 10^5^ cells/mL) and plated into 48-well plates (Nunclon Δ-surface, Nunc, Wiesbaden, Germany) for further analysis. The cells were then cultured for 72 h (95% air and 5% CO_2_, 100% humidity, 38 °C; Heraeus BB-6060, Hanau, Germany) to allow them to attach the cells to the bottom of the wells. The cells were then washed with medium (M199 with 0.1% BSA) and incubated in DMEM/HAM-12 + 0.1% BSA (culture medium). The measurement of viability was determined immediately after the incubation was terminated.

### 2.3. Preliminary Experiment: Effect of Pesticides on Cell Viability

Cervical tissues (n = 4 cows) were incubated for 72 h with each of the pesticides tested separately (at a dose of 10 ng/mL) or with E2 (10^−8^ M). Actinomycin D (Act D; 500 ng/mL), an inhibitor of RNA synthesis, and dimethyl sulfoxide (DMSO; 10%) were used as positive controls. Each treatment was performed in triplicate.

### 2.4. Experiment: Effect of Pesticides on Cervical Smooth Muscle Contractility

The cervical strips were incubated (24 or 48 h) without treatment (control), with E2 (10^−8^ M, as positive control; n = 4) or with the following treatments, which were studied separately: atrazine (n = 4), carbaryl (n = 4), cypermethrin (n = 4), DDT (n = 5), fenvalerate (n = 4), glyphosate (n = 5), malathion (n = 5) and thiram (n = 5), each at a dose of 10 ng/mL. This effective dose was chosen according to Wrobel and Mlynarczuk [59] and Wrobel et al. [64,65,66].

### 2.5. Determination of Cell Viability

Cell viability after pesticide treatment was measured using by a TOX-1 test (an in vitro toxicology assay kit based on MTT) according to the manufacturer’s protocol. This method is based on the ability of the mitochondrial enzyme dehydrogenase in living cells to convert a tetrazolium salt (MTT; yellow colour) into a formazan (blue colour). The cervical cells were incubated with MTT (20 µL/well) for 4 h. The absorbance of the reaction product was than measured at λ = 570 nm using an ELISA plate reader (Multiscan EX, Labsystem, Vantaa, Finland).

### 2.6. Measurement of Smooth Muscle Contractions

The strips were placed individually into the chambers of an HSE Schuler organ bath apparatus (March–Hugstetten, Germany) containing KRS (10 mL) consisting of NaCl (120.3 mM), KCl (5.9 mM), CaCl_2_ (2.5 mM), MgCl_2_ (1.2 mM), NaH_2_PO_4_ (1.2 mM), NaHCO_3_ (15.5 mM) supplemented with glucose (11.5 mM). Each strip was tied to the base and to the isometric contraction transducer (HSE Type 372) using a stationary hook and surgical silk, respectively. The KRS solution was maintained at 37.5 °C and was kept oxygenated (95% O2 and 5% CO_2_). All specimens were allowed to equilibrate for 2 h. Next, the forces of spontaneous isometric contractions of cervical smooth muscle were measured every 2 s for 15 min, in accordance with Wrobel and Mlynarczuk [59] and Wrobel et al., [64,65,66].

### 2.7. Statistical Analysis

The mean (±SEM) values of contraction force were expressed in mN and calculated from all measurements taken at four-second intervals for 15 min. All means (±SEM) were compared using one-way ANOVA for repeated measures, followed by the Newman-Keuls test after testing for normality. All statistical analyses and figure generation were performed using Prism 10 software (GraphPad Software, Inc., Boston, MA, USA).

## 3. Results

- The changes in cervical contractions were not evoked by the cytotoxic effects of the applied pesticides.
- Carbaryl, cypermethrin, thiram, and glyphosate increased the force of contraction of cervical strips.
- The administration of fenvalerate and E2 reduced in the intensity of cervical contractions.

**Figure 1:**
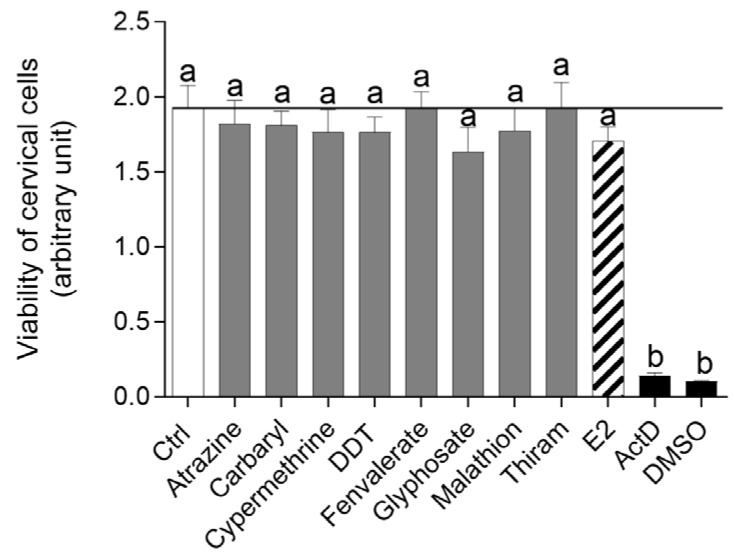
The mean (±SEM; n = 4 animals) viability of cervical cells after incubation (72 h) with the investigated pesticides (each at dose 100 ng/mL) and oestradiol (E2; 10^−8^ M). Actinomycin D (Act D; 500 ng/mL) and DMSO (10%) were used as positive controls. ^a,b^ (*p* < 0.01).

**Figure 2:**
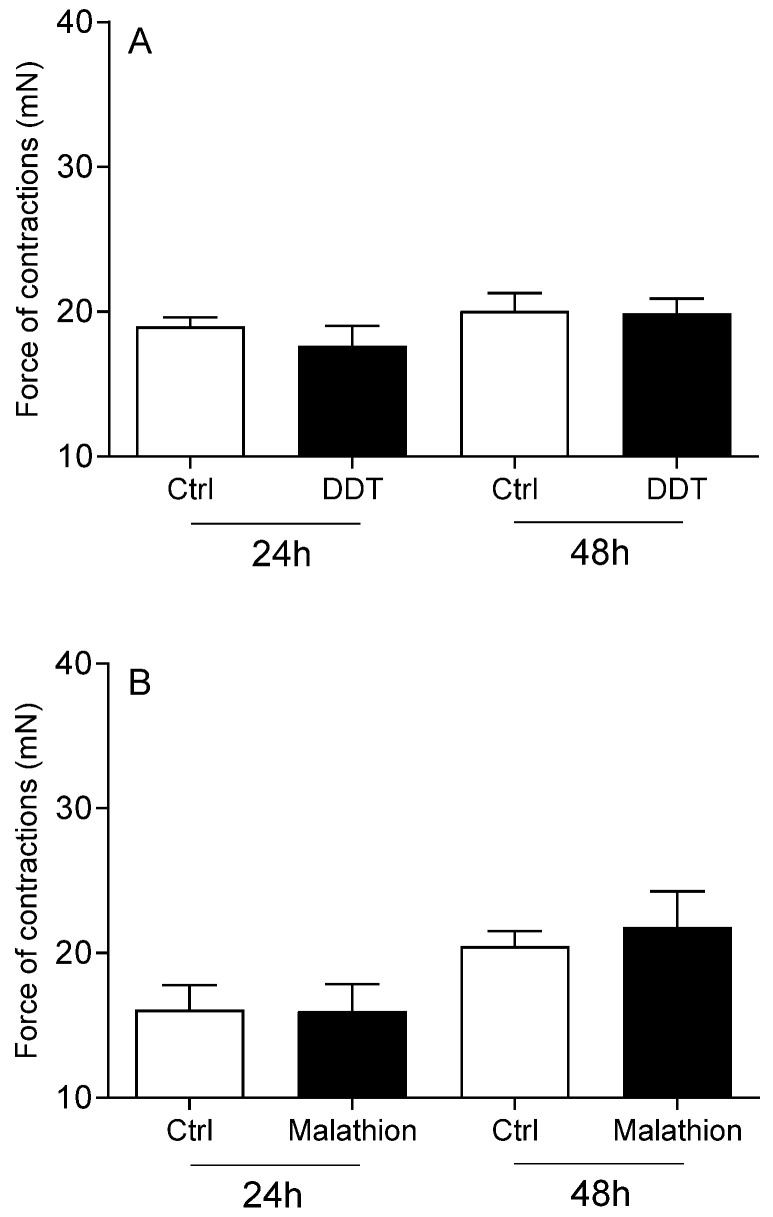
The mean (±SEM) force of the longitudinal muscle strips from the cervix after incubation (24/48 h) with DDT ((**A**) n = 5 animals) or malathion ((**B**) n = 5 animals), each at a dose of 10 ng/mL.

**Figure 3:**
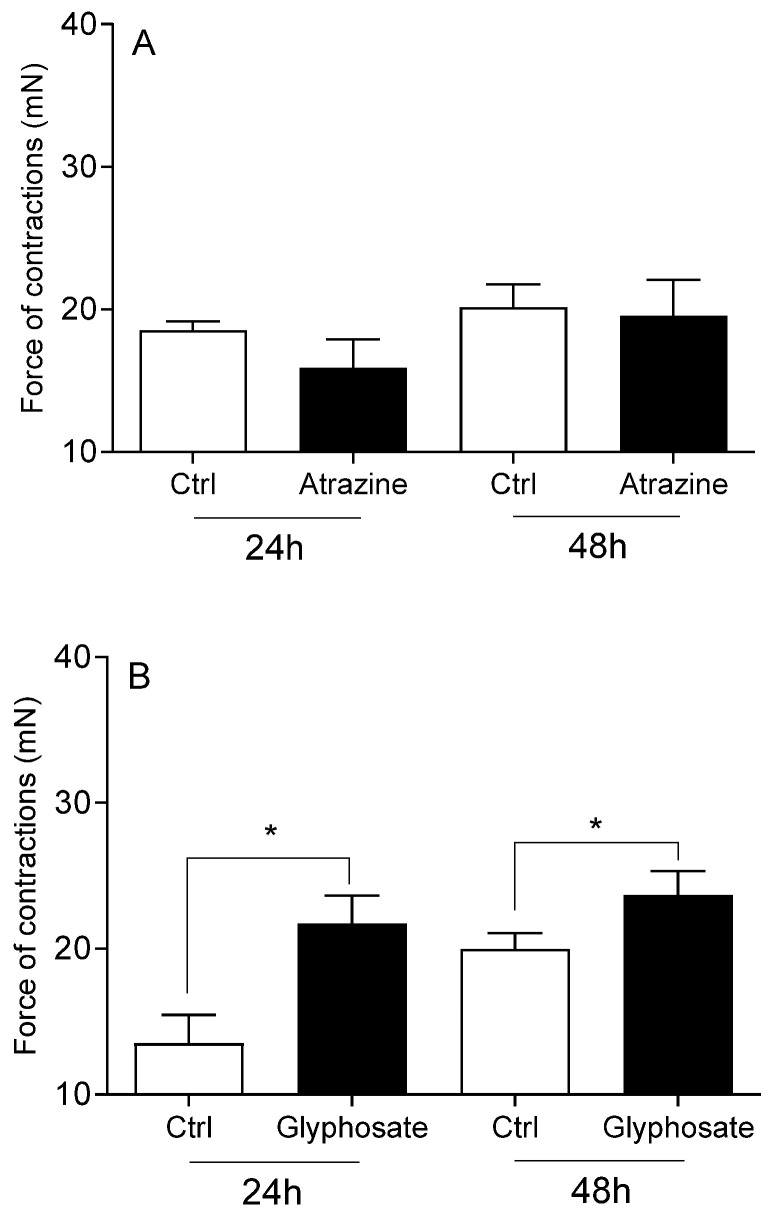
The mean (±SEM) force of the longitudinal muscle strips from the cervix after incubation (24/48 h) with common herbicides: atrazine ((**A**) n = 4 animals) or glyphosate ((**B**) n = 5 animals), each at a dose of 10 ng/mL. * (*p* < 0.05).

**Figure 4:**
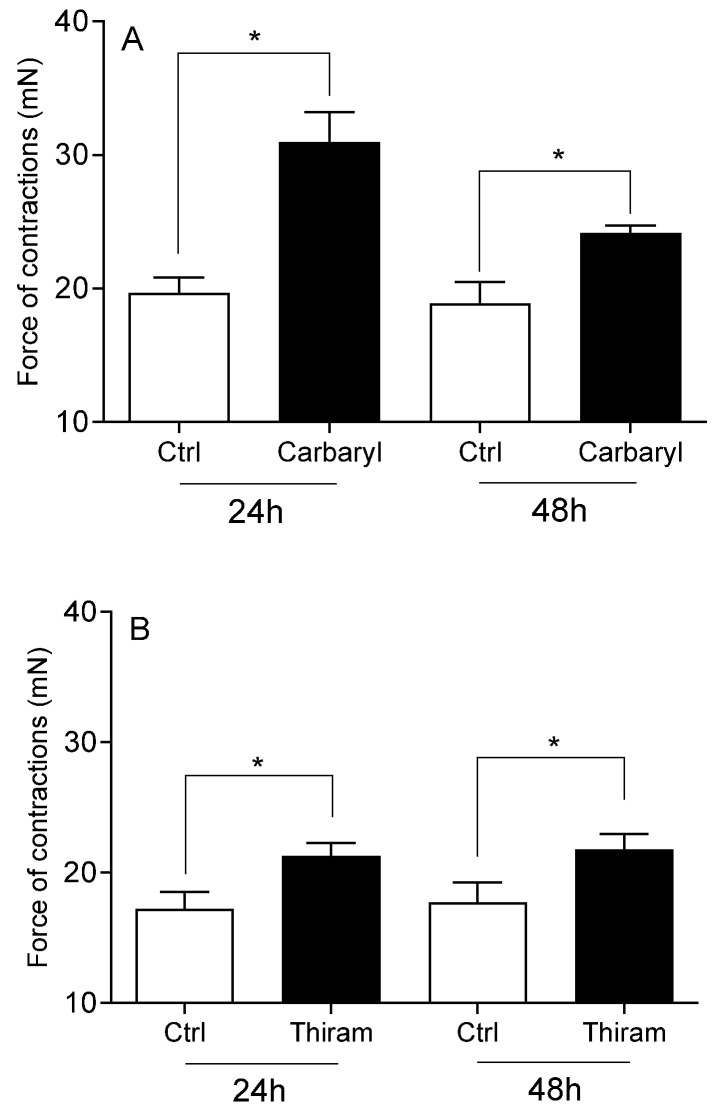
The mean (±SEM) force of the longitudinal muscle strips from the cervix after incubation (24/48 h) with carbamates: carbaryl ((**A**) n = 4 animals) or thiram ((**B**) n = 5 animals), each at a dose of 10 ng/mL. * (*p* < 0.05).

**Figure 5:**
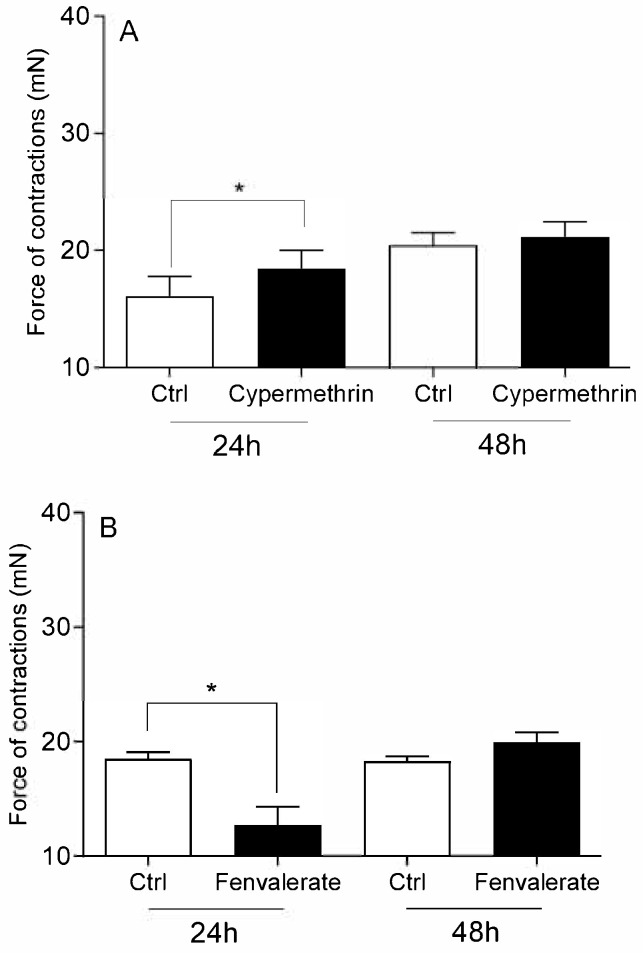
The mean (±SEM) force of the longitudinal muscle strips from the cervix after incubation (24/48 h) with pyrethroids: cypermethrin ((**A**) n = 4 animals) or fenvalerate ((**B**) n = 4 animals), each at a dose of 10 ng/mL. * (*p* < 0.05).

**Figure 6:**
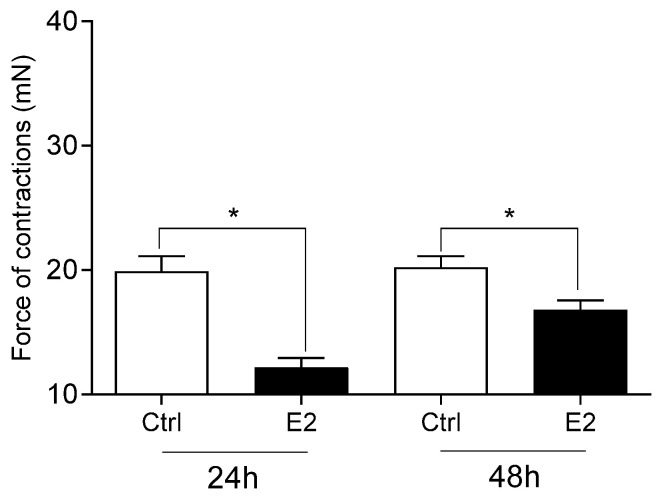
The mean (±SEM) force of the longitudinal muscle strips from the cervix after incubation (24/48 h) with oestradiol (E2, 10-8 M; n = 4 animals). * (*p* < 0.05).

The viability of cervical cells was unaffected by any of the studied pesticides or E2 (*p* > 0.05), compared to the vehicle DMSO and Act D (*p* < 0.01; Figure 1). Neither DDT (Figure 2A), malathion (Figure 2B) nor atrazine (Figure 3A) affected the force of cervical strip contraction (*p* > 0.05) after 24 h (37.5 °C) and 48 h (4 °C). However, carbaryl (Figure 4A), thiram (Figure 4B) and glyphosate (Figure 3B) increased (*p* < 0.05) it. Cypermethrin (Figure 5A) increased it (*p* < 0.05), whereas fenvalerate (Figure 5B) only decreased (*p* < 0.05) the force of cervical strip contraction after 24 h (37.5 °C). However, both pyrethroids had no effect (*p* > 0.05) after 48 h (4 °C). However, E2 decreased the force of cervical contractions (*p* < 0.05) in both conditions studied (Figure 6).

## 4. Discussion

We have carefully studied the mechanism by which the use of pesticides adversely affects cervical function in cattle in vitro during the oestrus cycle [59,64,65,66]. Therefore, we can assume that they have the potential to disrupt the onset of pregnancy. However, to estimate their effect on reproductive success and ultimately on birth, their direct effect during pregnancy must also be measured. In this context, the effect of the studied pesticides on the motor activity of the bovine cervix is particularly evident. To the best of our knowledge, this is the first report to present this information. It should also be mentioned that preliminary studies confirmed that the viability of bovine cervical cells was not unaffected by the studied dose of pesticides during pregnancy. This effective and environmentally relevant dose was previously selected according to data obtained during the oestrous cycle [59,64,65,66]. In addition, we compared the different treatment conditions, which may be useful for further studies of foreign substances acting on smooth muscle in an uncontrolled and prolonged manner.

The present study set out to investigate the hypothesis that glyphosate exerts a direct effect on the force of cervical contractions during pregnancy. The results of the study demonstrate that glyphosate does indeed have a direct effect on the force of cervical contractions during pregnancy. This is the first instance in which the direct effect of glyphosate on the spontaneous motor activity of the bovine uterus has been observed. Our previous studies have demonstrated that glyphosate does not affect the force of cervical [65] and myometrial [63] contractions during the oestrous cycle. This finding lends further support to our previous assertion that glyphosate cannot be designated as a direct risk factor for miscarriage or preterm labour. Conversely, it has been hypothesised that glyphosate may potentially induce significant cervical canal narrowing, thereby increasing the risk of delayed labour. This predicament, naturally, has the potential to present a considerable problem, particularly in the context of wildlife, where the absence of veterinary care is a salient issue.

It was demonstrated that both carbamates, carbaryl and thiram, produced a stimulatory effect on cervical contractions similar to that previously observed in strips treated with glyphosate. However, it was only thiram that produced the same effect on cervical motor function during the oestrous cycle, in contrast to carbaryl [59]. This confirms that the fungicide should not be mentioned as a potential risk factor for preterm birth, as it has been demonstrated to result in cervical canal closure. However, the effect of carbaryl is not so obvious. It has been demonstrated that during the oestrous cycle, carbaryl decreased the strength of cervical contractions [59], which may also affect the barrier preventing bacterial invasion of the uterine cavity. Nonetheless, this may be subject to alteration during pregnancy, which is characterised by a delay in labour.

Furthermore, cypermethrin has been demonstrated to augment the intensity of cervical contractions during pregnancy, though it exerts no direct influence on smooth muscle activity during the oestrous cycle [66]. In this respect, it can be hypothesised that this pyrethroid may exert its effect on labour in a manner analogous to that of the carbamates which were the subject of the study. On the other hand, fenvalerate has the unique property of relaxing the cervical muscles specifically during pregnancy. This indicates that it is the sole agent capable of directly preparing the cervix to soften and dilate prior to labour, a process that can result in the premature expulsion of the foetus. Furthermore, it has been demonstrated that this phenomenon serves to weaken the barrier against bacterial and fungal infiltration into the uterine cavity, thereby increasing the likelihood of endometritis. Surprisingly, fenvalerate has been observed to induce significant increases in cervical contractions in bovines during the oestrous cycle [66]. These contractions can result in the closure of the cervical canals and subsequent restriction of access to the uterine cavity. Consequently, it can be hypothesised that the substance has the capacity to impede fertilisation. Nevertheless, in accordance with the observations that have been conducted to date, it can be hypothesised that fenvalerate may also be a contributing factor to miscarriages.

In the present study, it was observed that some pesticides did not elicit a response. The representative of the organochlorine (DDT) and organophosphate (malathion) insecticides as well as the herbicide atrazine, did not affect the cervical contractions under either condition studied. As previously indicated, atrazine has been demonstrated to exert a significant impact on the force of cervical contractions [65] and opposite to myometrial contractions [62] during the oestrous cycle. Consequently, its effect tends to be unpredictable. Therefore, in order to provide a final assessment of the effect of atrazine on reproductive success, it is also necessary to measure myometrial contraction under atrazine treatment during pregnancy. In a similar manner, the administration of the same dosage of DDT and malathion was found to inhibit the force of contractions of the bovine cervix during the oestrous cycle [64] and of the rat aorta [68]. In addition, DDT or its metabolite (DDE) has been observed to stimulate the force of the contractions of both pregnant and nonpregnant uterine horns in cows [41,60,63] or rats [49,69] and bovine oviducts [70]. Therefore, the effect of DDT/DDE, which has exhibited a correlation with an increased risk of miscarriage [26,29,30] and preterm birth [24,27,28,31,32], is more likely to be caused by direct action on the myometrium than on the cervix. Despite the fact that malathion did not demonstrate the capacity to result in the cervical retention of the foetus in the uterus during pregnancy, it is not possible to incorporate its potential risk factor of decreased gestational duration, which was previously suggested [64,71]. Verification of the myometrial effect is also required.

It was also noted that E2 led to a reduction in the strength of cervical contractions during pregnancy. The observed decrease in cervical counterattraction has been shown to induce cervical relaxation [47,48]. This was suspected because plasma oestrogen levels are elevated during parturition in cows [38] and have been demonstrated to induce the production of prostaglandins [47,48]. Therefore, the cervix undergoes a series of changes leading to its softening, shortening and dilation at term [72]. In contrast, the bovine cervix remains closed during pregnancy, which is associated with a decline in E2 and an increase in P4 [73,74]. Therefore, the impact of any oestrogen-like substances during the mid-gestational period may be a crucial factor in the occurrence of cervical smooth muscle relaxation and preterm birth. However, the present study found that only fenvalerate was found to have such an effect. The remaining pesticides examined in this study exhibited either anti-estrogenic properties or no discernible effect. Despite the majority of these substances being classified as oestrogen-like, the present studies suggest that it is not possible to clearly attribute either an oestrogenic or anti-oestrogenic effect to the pesticides.

In our previous reports, we investigated the adverse effect of chlorinated pesticides [41,61,66] or polychlorinated biphenyls [75] on bovine myometrial contractions, which was prolonged to 48 or 72 h at a cold temperature of +4 °C. However, the effect of pesticides on cervical contraction during the oestrous cycle was only measured after 24 h at 38 °C, since it was not possible to undertake a longer incubation under these conditions [59,64,65,66]. Therefore, a comparative analysis was conducted to assess the efficacy of prolonging the effect of treatment and to evaluate the impact of different conditions. The two carbamates studied, as well as the two herbicides and representatives of chlorinated and phosphoorganic insecticides, demonstrated a comparable effect under both conditions. As is the case with E2, which also exerts an effect on cervical contractions in a similar manner in both conditions that have been studied. This finding serves as a confirmation of the conclusions drawn from the present study. However, two pyrethroids studied exerted their effect after a short treatment time at physiological temperature of cow, while this effect was inhibited at cold temperature after a longer incubation time. It is hypothesised that a 24 h period under physiological conditions is a sufficient timeframe for the study of the prolonged effect of xenobiotics on cervical contractions.

This study has several important limitations that need to be acknowledged. First, the results were obtained using an in vitro cervical smooth muscle model, which does not allow direct extrapolation to clinical reproductive outcomes such as miscarriage, preterm birth, or delivery disorders. Second, only parent pesticide compounds were investigated, whereas their biologically active metabolites were not included; future research should therefore address the potential effects of relevant metabolites. Third, the experiments were conducted using a single effective concentration, selected on the basis of earlier data and published studies, which ensures relevance but limits the ability to establish full dose–response relationships. Finally, the present study focused exclusively on cervical smooth muscle, without assessing other reproductive tissues such as myometrium, endometrium, or placenta. This narrows the scope of interpretation and highlights the need for further studies in a broader reproductive context.

## 5. Conclusions

In conclusion, the carbamates studied (carbaryl, thiram), glyphosate and cypermethrin had a stimulatory effect on the strength of cervical contractions in mid-pregnancy. The effect of atrazine, DDT and malathion were found to be negligible. Conversely, fenvalerate was observed to induce a relaxation of cervical contractions in a manner similar to E2. The results of this study suggest that pesticides are more likely to induce cervical contractions and inhibit labour than they are to induce miscarriages. However, further molecular investigations are required to confirm these findings. Moreover, it is generally accepted that a 24-h incubation at physiological temperature is adequate for the study of the longer-term effects of xenobiotics on cervical contractions. However, the findings are limited to ex vivo cervical tissue tested at a single effective concentration of the parent compounds. Further molecular and functional studies should therefore address relevant metabolites, evaluate dose–response relationships across a range of environmentally relevant exposures, and extend observations to other reproductive tissues. Although a 24-h incubation at physiological temperature proved adequate for assessing pesticide effects under controlled conditions, these mechanistic ex vivo results should not be directly extrapolated to clinical reproductive outcomes without in vivo or epidemiological confirmation.

## Data Availability

The original data has been presented in the study.
